# The Impact of a Rosemary Containing Drink on Cognition and Mood: The Role of Eye Blink Dynamics

**DOI:** 10.3390/neurosci7010015

**Published:** 2026-01-17

**Authors:** Leigh Martin Riby, Dimana Kardzhieva, Sam Fenwick, Sophia Fowler, Mark Moss

**Affiliations:** Department of Psychology, Northumbria University, Newcastle NE1 8ST, UK; dimana2.kardzhieva@northumbria.ac.uk (D.K.); mark.moss@northumbria.ac.uk (M.M.)

**Keywords:** rosemary, eye blinks, dopamine, acetylcholine, arousal, mood, wellbeing, ex-Gaussian, P3a ERP, attentional control

## Abstract

Rosemary (*Salvia rosmarinus*) has been linked to improvements in psychological wellbeing through cholinergic mechanisms. However, this study investigated whether individual differences in eye blink rate (EBR) and blink variability (EBV), which are proxies of dopaminergic activity and attentional control, influence the cognitive and mood-enhancing properties of a rosemary-containing drink. Forty-eight healthy adults completed a three-stimulus odd-ball cognitive task under rosemary or control conditions, while vertical electrooculograms were recorded. Event-related brain potentials (ERPs) were also measured using the P3a component at the Cz scalp electrode as an additional index of dopaminergic activity. Subjective mood and arousal (alert, contented, calm) were collected pre- and post-task using Bond–Lader visual analogue scales. Reaction times during the task were modelled with ex-Gaussian parameters (μ, σ, τ). Rosemary ingestion led to increased alertness and contentedness following the task. Cognitive effects were moderated by blink metrics, with significant interactions between rosemary and blink metrics for mean reaction time μ and response variability σ. Rosemary also increased P3a amplitudes, indicative of dopaminergic contribution. The effects of rosemary on cognition and mood were moderated by individual blink profiles, indicating that baseline neurocognitive state plays a role. Although cholinergic accounts are well established, this study highlights the use of proxies of dopamine to investigate broader neurotransmitter involvement in rosemary’s enhancing properties.

## 1. Introduction

Rosemary is a herb native to the Mediterranean region that has long been associated with cognitive enhancement and psychological wellbeing. Historically, rosemary was used in Egyptian burial rituals and symbolised fidelity and remembrance in ancient Greek ceremonies, including weddings and funerals (see [1,2]). The association with memory and cognition is not purely symbolic. In Hamlet, Ophelia remarks, “There’s rosemary, that’s for remembrance” (4.5.175), reflecting early cultural intuitions of its mnemonic effects [3]. Its presence in such traditions, alongside evidence of physical and psychological benefits, has contributed to its reputation as a cognitive enhancer [4]. Carnosic and rosmarinic acid are two of the active ingredients of rosemary, which have known antioxidant and anti-inflammatory properties. These compounds support the immune system, offer relief from pain, and are linked to cardiovascular protection [5]. Rosemary’s anxiolytic and stimulatory effects have also been linked to reduced stress, improved emotional regulation, and, particularly relevant here, cognitive facilitation [6]. In the present study, we aim to identify the specific mood and attentional components that improve during cognitive task engagement and examine the moderating effects of dopamine-linked blink metrics.

Consider first acetylcholine (ACh), a neuromodulator central to attention and memory [7]. ACh is typically released from the basal forebrain, notably the nucleus basalis of Meynert, with widespread cholinergic projections targeting the cortex [8,9]. These projections support both synaptic and volume transmission, allowing broad modulation of the cortical activity thought to be involved in attentional control, including the regions of the frontoparietal and default mode networks [10]. The active compounds of rosemary, including 1,8-cineole and carnosic acid, have been shown to inhibit acetylcholinesterase (AChE) and may increase ACh availability [11]. This finding is consistent with the action of pharmaceutical cholinesterase inhibitors used in the treatment of Alzheimer’s disease [12]. Animal models also suggest rosemary may have anxiolytic and antidepressant effects alongside AChE inhibition properties [12]. These findings suggest cholinergic involvement. However, given rosemary’s influence on attentional and affective processes, examining dopaminergic pathways may offer a theoretically grounded and potentially worthwhile route to examine the enhancing properties of rosemary further.

In addition to involvement in emotional processing, dopamine plays a critical role in the regulation of attention and executive function, particularly in the filtering of distractions and the processing of novelty [13]. Disruptions in dopamine signalling in the prefrontal cortex and basal ganglia may impair a variety of aspects of attention functioning and behaviour (see [14] for a review). This neurochemical framework has informed a range of pharmacological interventions. For instance, psychostimulants such as methylphenidate and amphetamines, commonly used in the treatment of ADHD, enhance dopaminergic transmission and improve attentional ability [15]. Similarly, dopaminergic agents have been explored in cognitive enhancement studies targeting episodic memory and attention in clinical populations, including Parkinson’s disease [16] and schizophrenia [17]. Nutritional interventions have also demonstrated modulatory effects on dopamine. Tyrosine, a dietary precursor to dopamine, has been shown to influence cognitive performance under stress and fatigue [18]. Therefore, it is hardly surprising that compounds found in a variety of herbs, such as rosemary, may exert dopaminergic effects possibly by interacting with brain receptors. Specifically, rosemary’s antidepressant-like properties have been linked to activity at dopamine D1 and D2 receptors, as well as noradrenergic and serotonergic systems [19]. These interactions suggest a broader neuromodulatory profile that may contribute to its cognitive and mood-related effects. From a neuropsychological perspective, the eye blink rate (EBR) has emerged as a non-invasive proxy for central dopamine activity and is employed in the present study.

While the relationship is unclear, EBR has been shown to vary across clinical populations in ways that may reflect dopaminergic status. For example, elevated blink rates are often reported in schizophrenia, while reduced rates are typically observed in Parkinson’s disease [20], which is consistent with underlying dopamine dysregulation. Our previous work on the enhancing properties of music, mind wandering, and creativity (e.g., [21]), alongside studies elsewhere [22], has established the use of blink metrics as indicators of dopaminergic function, attentional control, and emotional processing. Importantly, the blink rate is sensitive to cognitive and motivational demands and may increase during internally focused states involved in self-generated thoughts [23,24]. Blink variability (EBV), though less commonly discussed, may reflect transient shifts in attentional engagement but is included due to the partial exploratory nature of the present work.

### The Current Study

The present study aimed to investigate the cognitive and mood-enhancing effects of rosemary ingestion, with a focus on the moderating role of eye blink metrics. Eye blinks were used as putative non-invasive proxies of dopamine function and attentional control, allowing us to explore individual differences in responsiveness to rosemary. Building on previous work linking rosemary to cholinergic mechanisms, this study examined whether dopaminergic tone influences cognitive performance and subjective mood. Reaction time data were analysed using ex-Gaussian modelling (Mu, Sigma, Tau) to provide a comprehensive examination of attentional engagement during task, which mirrors previous work on Stroop task performance [25]. In addition, the P3a ERP component was included as a secondary and confirmatory index of dopaminergic activity to aid in the interpretation of the blink-related findings. While P3a is often linked to dopaminergic activity, it may also reflect broader physiological aspects of dopamine function beyond baseline activity. These measures provide indirect information about dopaminergic activity and also reflect broader cognitive processes; so, any dopaminergic interpretation in the present study is necessarily cautious. By integrating behavioural and electrophysiological measures, the study aimed to clarify how rosemary interacts with baseline neurocognitive states to influence attention, arousal and mood. This work extends our earlier rosemary ERP findings [26] by testing whether these effects differ as a function of baseline blink patterns. By adding blink metrics and ex-Gaussian modelling, the study incorporates indirect proxy measures with cautious links to neuromodulatory influences, including dopaminergic contributions. It therefore examines whether rosemary influences cognition and mood in a state-dependent way.

## 2. Methods

### 2.1. Participants

Forty-eight adults participated in the study (32 women; mean age = 30.7; range 18–63; SD = 11.2). All participants were right-handed and reported no history of neurological disorders. Screening excluded individuals with a current diagnosis of depression or anxiety, substance use, migraines, or any of the following: anaemia, heart disorder, high blood pressure, respiratory disorder, diabetes, pregnancy, history of seizures, or current use of prescribed, illicit, or herbal medication. Participants with food allergies or sensitivities were also excluded. Allocation to treatment condition was randomised prior to testing. Ethical approval was granted by the Department of Psychology Ethics Board at Northumbria University (Reference-882), and all procedures were conducted in accordance with the Declaration of Helsinki. Written informed consent was obtained from all participants. Further details and a description of part one of this program of work can be found in [26].

### 2.2. Treatment

Participants consumed either 330mL of rosemary water or plain still water. The rosemary drink was supplied by No1 Botanicals and contained both a volatile oil extract and a hydrolat derived from fresh rosemary via steam distillation. The extract included terpenes such as 1,8-cineole (0.025 mg/mL) and rosmarinic acid (0.13 mg/mL), while the hydrolat contained lower levels of terpenes (1,8-cineole at 0.012 mg/mL) and no rosmarinic acid. Production and analysis were carried out by Blue Sky Botanics, Castle Farm, Upton Bishop, Ross-on-Wye, UK.

To maintain blinding, participants were informed that both drinks had been matched for taste. Those in the rosemary condition were told the placebo had been flavoured to match, while those in the control condition were told the rosemary extract had been taste-neutralised. The researcher was also blind to condition allocation.

## 3. Measures

### 3.1. The 3-Stimulus Odd-Ball Task

Participants completed a 3-stimulus visual odd-ball task designed to assess attentional control [27]. Stimuli were presented using E-Prime software (version 2.0) on a 17.5-inch monitor. The task involved discriminating between three types of stimuli: a frequent standard stimulus (green square, area = 16 cm^2^; 74% of trials), a rare target stimulus (red circle, area = 12.6 cm^2^; 13% of trials), and a novel distractor stimulus (green square, area = 256 cm^2^; 13% of trials). Each stimulus was displayed for 100 ms, followed by a variable interstimulus interval between 830 and 930 ms. Participants responded to target stimuli using the spacebar on a PC keyboard. A short practice block preceded the main task to ensure participants understood the nature of the task.

### 3.2. Electrooculograms (Eye Blinks)

Vertical electrooculograms (VEOG) were recorded using electrodes placed above and below the eye to capture blink activity. Participants were asked to minimise excessive blinking and movement during the task. Researchers responsible for blink identification were trained in the characteristics of ocular EEG data, including blink duration, waveform shape, and tempo. The eye blink rate (EBR) was calculated as the total number of blinks occurring during the 8-min odd-ball task. The eye blink variability (EBV) was assessed by dividing the task into eight 60 s intervals and calculating the standard deviation of blink counts across these intervals. Although blink counts are not assumed to be normally distributed, the standard deviation across time bins provides a descriptive index of within-task fluctuations in attentional stability.

### 3.3. Event-Related Potentials

Although ERP data were recorded during the task, only the P3a (measured at the Cz scalp electrode) was used as a secondary dopamine metric, as the current paper focuses on blink metrics, subjective arousal, and cognition. A Biosemi electrode cap (https://www.biosemi.com (accessed on 24 December 2025)) with 32 channels of EEG recordings was employed to measure ERPs. Signals were digitised at a rate of 2048 Hz. The CMS (Common Mode Sense electrode; placed centre between Cz and C3 electrodes) and DRL (Driven Right Leg electrode; placed between the Cz and C4 electrodes) were used as the “ground” electrodes (see https://www.biosemi.com). The EEG recordings were referenced using the average electrode method. Signals were band-pass-filtered at 0.46–30 Hz during the acquisition. Offline processing (Neuroscan Edit 4.5, Compumedics, El Paso) comprised the following: automatic eyeblink correction (regression-based ocular artefact reduction algorithms), artefact rejection (values outside the range −75 uV to 75 uV), and baseline correction using the pre-stimulus interval (−200 ms). Epoching of the continuous EEG files was then performed using stimulus-triggered epochs (−200 to 1000 ms). The measurement intervals were selected based on visual inspection of the grand-average ERPs (P3a 350–470 ms) and on reported time intervals in our previous research using this paradigm and elsewhere (see [26] for a discussion). The P3a typically shows maximal fronto-central amplitude, consistent with our previous work using this paradigm. Average amplitudes were calculated in this range for the P3a at CZ.

### 3.4. Mood and Arousal Measures

Subjective mood and arousal were assessed using the Bond–Lader visual analogue scales. This measure comprises a series of bipolar items presented as 100 mm horizontal lines anchored by opposing adjectives (e.g., “alert–drowsy”, “calm–excited”, “contented–discontented”). Participants were asked to mark a point along each line that best represented their current state. Ratings were collected immediately before and after completion of the odd-ball task. Scoring involved the grouping into three composite dimensions: alertness, calmness, and contentedness. These composites were derived by averaging relevant items within each dimension, as outlined in the original Bond–Lader methodology [28]. The resulting scores were used to examine changes in subjective state across the testing session and to evaluate potential treatment effects.

## 4. Results

### 4.1. Subjective Arousal and Mood Ratings

The participants completed ratings of their alertness, calmness, and contentedness before treatment and after the task. Paired-samples t-tests (1-tailed) were conducted to assess pre–post changes. In the rosemary condition, there was a significant increase in alertness from pre- to post-task (t(22) = −2.41, *p* = 0.013). Participants also reported feeling significantly more content after the task compared to baseline (t(22) = −2.63, *p* = 0.008). There was no significant change in calmness. In the control condition, no significant changes were observed for alertness or calmness. However, there was a significant increase in contentedness, with scores rising from pre- to post-task (t(22) = −1.97, *p* = 0.031). Overall, these results indicate that rosemary significantly enhanced subjective alertness and contentedness, while the control condition only showed a modest improvement in contentedness, likely due to task completion effects. The treatment effects are summarised in Table 1. One-tailed tests were used for mood outcomes, because increases in alertness and positive affect following rosemary exposure were predicted a priori based on the previous literature.

### 4.2. Blink Metrics (Total Rate and Variability)

**Figure 1:**
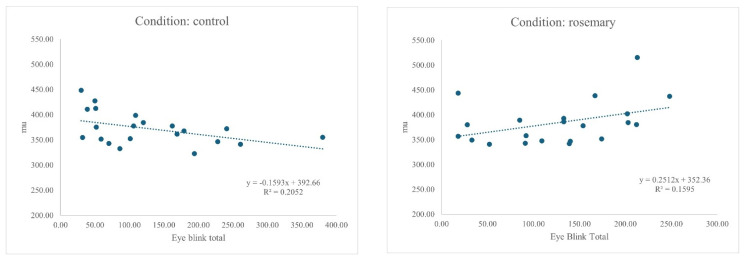
Relationship between EBR and Mu (μ) for control and rosemary conditions. Final figures and tables are supplied as a separate file to aid production; embedded versions are retained in the manuscript for reference.

An ANCOVA was conducted to examine whether the effect of the condition (rosemary vs. control) on the mean response time μ was moderated by individual differences in EBR. The overall model was significant, F(3, 38) = 3.08, *p* = 0.039, partial η^2^ = 0.20. There was a trend-level main effect of condition, F(1, 38) = 3.52, *p* = 0.068, partial η^2^ = 0.09. The interaction between the condition and blink rate was significant, F(1, 38) = 8.08, *p* = 0.007, partial η^2^ = 0.18, 95% CI [0.02, 0.39], indicating that the relationship between blink rate and mu differed depending on the condition. Specifically, higher blink rates were associated with faster responses in the control group and slower responses in the rosemary group, reflecting a reversal in slope across conditions (see Figure 1).

**Figure 2:**
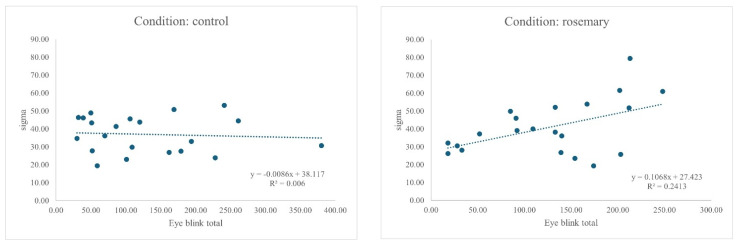
Relationship between EBR and sigma (σ) for control and rosemary conditions. Final figures and tables are supplied as a separate file to aid production; embedded versions are retained in the manuscript for reference.

An ANCOVA was conducted to examine the effect of the aroma drink condition (control vs. rosemary) on the response time variability (σ), with EBR entered as a continuous between-subjects factor. The interaction between the condition and blink rate was significant, F(1, 38) = 4.69, *p* = 0.037, η^2^ = 0.110, 95% CI [0.00, 0.31], indicating that the relationship between the blink rate and σ differed by condition (see Figure 2), with the association restricted to rosemary.

A further ANCOVA revealed that the main effects of the condition and blink rate, as well as their interaction, were not significant, indicating no meaningful differences in τ (reflecting lapses of attention; [24]) across conditions or as a function of blink rate. Similarly, an ANCOVA examining the proportion of hits revealed no significant main effect of treatment nor a significant interaction with the blink rate.

To explore whether the P3a amplitude (measured at Cz scalp electrode) was associated with the blink rate, an ANCOVA was conducted. The main effect of condition approached significance, F(1, 34) = 3.77, *p* = 0.061, η^2^ = 0.10, with higher P3a amplitudes in the rosemary condition.

**Figure 3:**
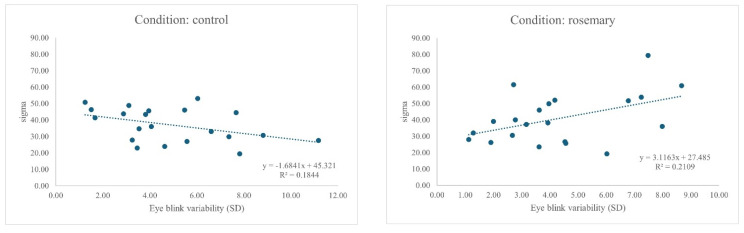
Relationship between EBV and sigma (σ) for control and rosemary condition. Final figures and tables are supplied as a separate file to aid production; embedded versions are retained in the manuscript for reference.

The next series of ANCOVAs considered EBV. When considering mu, there were no significant main effects or interaction effects observed for the mean response time μ when including blink variability in the model. An ANCOVA revealed a significant main effect of the condition on σ, F(1, 38) = 4.92, *p* = 0.033, η^2^ = 0.115, 95% CI [0.00, 0.32], with greater response variability in the rosemary condition than in the control. Importantly, a significant interaction between the condition and blink variability emerged, F(1, 38) = 4.83, *p* = 0.013, η^2^ = 0.203, 95% CI [0.00, 0.32], indicating that the effect of the drink on σ depended on individual differences in blink variability (see Figure 3).

An ANCOVA examining τ revealed no significant main effect of the condition nor a significant interaction with the blink variability. Similarly, the ANCOVA examining the hit rates and the P3a amplitude both revealed no significant main effect of the condition nor a significant interaction with blink variability.

### 4.3. Bivariate Correlations

Pearson’s bivariate correlations were computed to assess the relationships between all variables, as shown in Table 2 and Table 3.

Data were examined prior to analysis to ensure there were no apparent data anomalies.

## 5. Discussion

The present study tested not only the domains enhanced by rosemary but also whether these benefits depend on individual differences in spontaneous blinking, a proxy for dopaminergic tone and attentional control. By combining ex-Gaussian modelling of response times, subjective mood ratings, and electrophysiological measures of attentional orienting (P3a), we aimed to clarify whether rosemary exerts broad effects or whether its influence is better understood as state-dependent. The central finding was that rosemary’s effects are not uniform across individuals but depend on blink profiles, supporting a state-dependent model of cognitive enhancement. These findings extend our earlier ERP work on rosemary [26], which focused solely on neural markers. The present study shows that the behavioural and mood effects of rosemary vary with baseline blink profiles, indicating a more state-dependent pattern of enhancement.

The results revealed that rosemary ingestion significantly enhanced subjective alertness and contentedness, consistent with previous behavioural and neurophysiological investigations (e.g., [29]). These changes occurred from baseline to post-task, indicating that rosemary promoted mood facilitation during active cognitive engagement. The control drink also produced a modest increase in contentedness, likely due to task completion and satisfaction, but it did not influence alertness, which underscores the specificity of rosemary’s impact. Rosemary has traditionally been associated with cholinergic mechanisms, with active constituents such as 1,8-cineole and rosmarinic acid inhibiting acetylcholinesterase and increasing cholinergic signalling. These mechanisms are well established in supporting memory and attentional processes. However, the observed enhancement in subjective alertness and contentedness may reflect broader arousal mechanisms, including increased sympathetic nervous system activity (see [30]). Indeed, rosemary and other botanicals such as peppermint [31] and citrus oils [32] have been shown to increase arousal and alertness. Recent longitudinal findings concur and suggest that nightly exposure to rotating botanical aromas, including rosemary, modulates central arousal pathways, potentially via noradrenergic and limbic system engagement [33]. The current findings support this on a self-report level, and future work can confirm whether the subjective experience coincides with central arousal activation.

The main consideration in the present work was the moderating effect of blink dynamics. Our ANCOVAs revealed a significant rosemary × blink rate interaction for μ and σ, indicating that individual differences in the blink rate moderate rosemary’s effects on response speed and variability. Specifically, participants with higher blink rates showed slower and more variable responses under rosemary, possibly reflecting enhanced dopaminergic sensitivity. The reversal in the blink–μ slope pattern may reflect two overlapping processes. First, individuals with lower blink rates, potentially indexing reduced dopaminergic tone, may have had a larger scope for improvement, consistent with inverted-U models of neuromodulation and enhancement (e.g., glucose facilitation; [34]). While a higher blink rate is associated with greater cognitive flexibility [35], its relationship with performance follows an inverted U-shaped curve, where an extremely high blink rate is linked to distractibility and an extremely low blink rate to perseveration [36]. Second, the crossover pattern does not reflect impaired performance but rather a shift in cognitive strategy. As slower responses can accompany more diffuse or exploratory attentional states, rosemary may have modulated the balance between focused and flexible cognition, particularly in high-blink individuals. Riby and colleagues (e.g., [26]) argue that such profiles may reflect increased engagement with task-irrelevant as well as relevant information. Previous evidence supports the notion that the blink rate reflects modulation between task-oriented and default-mode attention allocation, reflecting updating of information and transitions between attentional phases [37]. Rosemary may therefore modulate attentional control not by enhancing speed or consistency but by altering the balance between focused and diffuse cognitive states. No significant interactions were observed for τ or the hit rate, reinforcing the specificity of these blink condition effects. Importantly, the effects were selective to σ rather than τ. τ is typically associated with infrequent attentional lapses and extreme slow responses, σ reflects variability around the central portion of the response time distribution and is more closely linked to fluctuations in executive control. The absence of comparable τ effects therefore suggests that rosemary influenced attentional stability rather than reducing occasional lapses.

Both the blink rate and P3a relate to dopaminergic activity, but they are not specific indicators; so, interpretations of dopaminergic involvement should remain cautious. While the blink rate is the better-established marker of dopaminergic function, EBV is now recognised as a valuable measure in its own right. Considering that the overall blink rate has complex temporal relationships with task phases [38], it is valuable to account for blink variability. In the current data, the blink variability moderated the effect of rosemary on σ only. It demonstrated that individuals with higher EBV exhibited increased response variability in the rosemary condition, consistent with a shift toward more flexible attentional control. EBV reflects fluctuations in control and arousal, providing an index of attentional stability over time. A key demonstration comes from [39], who showed that the resting EBV was positively correlated with IQ, and high-IQ participants displayed greater variability than those with lower scores. Our study builds on this by demonstrating that rosemary’s effects are moderated by the baseline EBV, while more broadly advancing the use of blink metrics in botanical and olfactory cognitive neuroscience. Considering rosemary’s involvement in attention regulation through acetylcholine availability, exploring EBV provides relevant insight into the complexity of attentional mechanisms.

Correlational analyses (Table 2 and Table 3) showed consistent links between blink measures, mood, and task performance across both treatment conditions. Though exploratory, these patterns complement the ANCOVA results and suggest that rosemary modulated how blink behaviour relates to attentional control. Overall, however, given the number of analyses conducted, particularly with the exploratory correlations, the findings should be interpreted cautiously. In addition, full blinding was unlikely because the rosemary drink had a distinct taste and aroma relative to the water control, and expectancy effects cannot be entirely ruled out.

The electrophysiological data offer further support for a state-dependent interpretation. Rosemary ingestion was associated with increased P3a amplitude at Cz, a component linked to involuntary attentional orienting and novelty detection. This interpretation aligns with integrative accounts of the P300 component. Polich’s framework distinguishes P3a and P3b and highlights P3a’s sensitivity, with its fronto-central distribution, to neuromodulatory influences on attentional orienting and cognitive control. Dopaminergic activity is viewed as one of several interacting contributors within this system, such that P3a provides a non-specific indirect index of neuromodulatory engagement rather than a selective dopamine marker. Polich’s integrative model ([27]; see also [40]) for a recent comprehensive review highlighting the non-specific and multi-neurotransmitter influences on P3 components] attributes P3a to frontal–central engagement with salient stimuli, modulated by dopaminergic activity in prefrontal and cingulate regions. Its amplitude varies with neurochemical state, task relevance, and individual differences in control. Reduced P3a is observed in conditions marked by dopaminergic dysfunction (e.g., Parkinson’s, schizophrenia), while elevated amplitudes have been linked to novelty seeking and intact executive function. In this light, rosemary’s enhancement of P3a may reflect increased dopaminergic modulation of attentional orienting, particularly in individuals with greater baseline flexibility as indexed by the EBV. Notably, the P3a amplitude did not correlate with either EBR or EBV (see Table 2 and Table 3), supporting the view that blink metrics and ERP components index distinct facets of neuromodulatory function. Blinks likely reflect tonic attentional readiness over extended intervals, while P3a captures phasic activity. This is consistent with the evidence that blink rates, unlike P3a, are not stimulus-dependent and reflect broader primary consciousness phenomena and attention mode modulation [37]. This dissociation underscores the value of convergent physiological markers in characterising individual responsiveness to botanical interventions.

The present findings underscore the importance of jointly considering behavioural performance and physiological responses when evaluating the cognitive effects of botanical interventions. As previously discussed, cognitive performance has a complex relationship with dopamine and, thus, blink rate as an indicator of dopaminergic activity. The current literature also focuses extensively on overall blink rates, leaving room for better understanding of how blink rate variability fluctuates in response to phases of attention, stimulus encoding, and exposure to interventions. Future research should also utilise working memory tasks to explore dopamine-modulated attention as a ‘gate-switching’ mechanism for memory encoding and consolidation. The eye blink rate has been linked to shifts between updating and maintenance mode during working memory performance [41]. In light of the current findings, rosemary may offer a low-cost accessible tool for flexibly engaging and disengaging task-oriented attention. In realistic, ecologically valid settings such as the workplace, attention fluctuates in relation to task type, rote work, and context [42]. As such, cognitive focus is vulnerable to habituation and fatigue, which is reflected in longer intervals between blinks [43]. Botanical interventions, such as rosemary, may be particularly useful for applied uses beyond the laboratory, where sustaining attention over prolonged periods would benefit from a flexible ability to shift between attentional states.

Lastly, the relationship between rosemary exposure and blink rates may help inform a wellbeing-oriented approach to attention in applied practice. In a laboratory context, mind-wandering and deviations in attention away from task stimuli lower performance. Indeed, mind-wandering is linked to decoupling attention from perceptual information and engaging with internal default-mode responses [44]. Task-unrelated thoughts are associated with higher eye blink rates, suggesting that increased dopaminergic activity corresponds to a diffuse centre of attention [24]. Thus, stable and highly concentrated attentional states may be difficult to sustain, while flexible and diffuse attention would permit shifting focus as needed, engaging and decoupling self-referential and task-oriented thought, thereby ensuring a smoother transition between states. In this context, the present findings motivate future research examining whether rosemary-related changes in attentional dynamics are associated with subjective state in more ecologically valid settings.

## 6. Conclusions

This study demonstrates that rosemary’s cognitive and mood-enhancing effects are state-dependent, varying systematically with individual blink profiles that index dopaminergic tone and attentional control. Theoretically, this extends existing cholinergic accounts by introducing a dual-neuromodulator framework, in which rosemary is proposed to influence both acetylcholine- and dopamine-related mechanisms of attention and arousal. At a broader neurochemical level, evidence linking rosemary bioactives to dopaminergic mechanisms is largely derived from non-human and preclinical models. Compounds such as rosmarinic and carnosic acid have been reported to indirectly influence dopaminergic signalling, alongside noradrenergic and serotonergic systems, rather than acting on dopamine synthesis or release per se (see, for example [45]). The integration of blink dynamics, ex-Gaussian modelling, and electrophysiological measures offers a novel and reproducible approach for examining how botanical interventions interact with baseline neurocognitive state in cognitive enhancement. By situating these effects alongside blink dynamics, the study advances beyond our previous rosemary ERP work. It offers a more integrated account of how rosemary interacts with the baseline neurocognitive state.

## Figures and Tables

**Table 1:** Means and standard deviations for key variables by condition (control vs. rosemary), with Cohen’s *d* effect sizes.

Measure	Control M (SD)	Rosemary M (SD)	Cohen’s *d*
Eye Blink Rate (EBR)	120.24 ± 90.21	122.91 ± 67.77	0.03
Blink Variability (EBV)	4.69 ± 2.52	4.14 ± 2.21	−0.23
Alertness (Pre)	35.11 ± 12.08	36.91 ± 11.56	0.15
Calmness (Pre)	30.91 ± 14.43	33.54 ± 17.75	0.17
Contentedness (Pre)	24.22 ± 9.48	26.67 ± 10.97	0.25
Alertness (Post)	38.32 ± 18.48	45.06 ± 18.01	0.37
Calmness (Post)	32.92 ± 13.99	32.87 ± 14.00	−0.00
Contentedness (Post)	28.34 ± 10.94	30.97 ± 15.06	0.19
Mu (μ)	372.04 ± 32.40	383.98 ± 44.06	0.31
Sigma (σ)	37.01 ± 10.15	40.87 ± 15.24	0.30
Tau (τ)	33.86 ± 12.74	36.81 ± 10.61	0.25
Hits	75.75 ± 21.53	78.37 ± 14.67	0.13
P3a Amplitude (Cz)	4.79 ± 2.86	6.44 ± 2.89	0.63

**Table 2:** Pearson’s correlations (control condition) between blink proxies (total and variability), alertness (alert, calm and content), cognitive performance (μ, τ, σ, and hits), and the P3a ERP (Cz scalp electrode). Final figures and tables are supplied as a separate file to aid production; embedded versions are retained in the manuscript for reference.

	1	2	3	4	5	6	7	8	9	10	11	12	13
1. Eye blink total	1	0.588 **	0.555 **	0.232	0.316	0.309	0.051	0.063	−0.453 *	−0.078	0.042	−0.281	0.176
2. Eye blink variability	0.588 **	1	0.462 *	0.164	0.302	0.416 *	0.174	0.243	−0.138	−0.429	−0.062	0.114	−0.319
3. Alertness (pre)	0.555 **	0.462 *	1	−0.117	0.249	0.692 **	−0.132	0.266	−0.296	−0.428	0.396	0.857	0.498
4. Calmness (pre)	0.232	0.164	−0.117	1	0.237	0.048	0.220	−0.049	0.084	−0.063	−0.342	0.162	−0.086
5. Contentedness (pre)	0.316	0.302	0.249	0.237	1	0.496 *	0.265	0.267	−0.312	−0.480 *	−0.061	−0.113	−0.040
6. Alertness (post)	0.309	0.416 *	0.692 **	0.048	0.496 *	1	−0.133	0.572 **	−0.146	−0.234	−0.330	−0.036	0.424
7. Calmness (post)	0.051	0.174	−0.132	0.220	0.265	−0.133	1	0.511 *	−0.197	−0.197	−0.438	0.205	0.446
8. Contentedness (post)	0.063	0.243	0.243	0.266	0.267	0.572 **	0.511 *	1	−0.269	−0.104	−0.400	−0.314	0.436
9. Mu (μ)	−0.453 *	−0.138	−0.296	0.084	−0.312	−0.146	−0.197	−0.269	1	0.242	0.136	0.448 *	−0.252
10. Sigma (σ)	−0.078	−0.429	−0.428	−0.063	−0.480 *	−0.234	−0.197	−0.104	0.242	1	−0.002	−0.250	0.262
11. Tau (τ)	0.042	−0.062	0.396	−0.342	−0.061	−0.330	−0.438	−0.400	0.136	−0.002	1	0.046	−0.163
12. Hits	−0.281	0.114	0.857	0.162	−0.113	−0.036	0.205	−0.314	0.448 *	−0.250	0.046	1	−0.559 *
13. P3a ERP	0.176	−0.319	0.498	−0.086	−0.040	0.424	0.446	0.436	−0.252	0.262	−0.163	−0.559 *	1

Note: * *p* < *0*.05, ** *p* < 0.01.

**Table 3:** Pearson’s correlations (rosemary condition) between blink proxies (total and variability), alertness (alert, calm and content), cognitive performance (μ, τ, σ, and hits), and the P3a ERP (Cz scalp electrode). Final figures and tables are supplied as a separate file to aid production; embedded versions are retained in the manuscript for reference.

	1	2	3	4	5	6	7	8	9	10	11	12	13
1. Eye blink total	1	0.7760 **	0.228	0.201	0.158	0.056	0.092	0.077	0.399	0.491 *	0.118	0.001	−0.227
2. Eye blink variability	0.776 **	1	0.215	0.262	0.426 *	0.221	−0.049	0.306	0.388	0.459 *	0.096	0.345	−0.310
3. Alertness (pre)	0.228	0.215	1	0.337	0.467 *	0.465 *	−0.275	0.475 *	0.242	0.388	0.104	−0.091	−0.251
4. Calmness (pre)	0.201	0.262	0.337	1	0.826 **	0.375	0.498 *	0.743 **	0.291	0.433	0.290	0.323	0.138
5. Contentedness (pre)	0.158	0.426 *	0.467 *	0.826 **	1	0.469 *	0.267	0.865 **	0.302	0.371	0.188	0.388	−0.095
6. Alertness (post)	0.056	0.221	0.465 *	0.375	0.469 *	1	−0.043	0.739 **	−0.038	−0.134	−0.117	0.195	0.015
7. Calmness (post)	0.092	−0.049	−0.275	0.498 *	0.267	−0.043	1	0.241	0.041	0.155	−0.149	−0.221	0.275
8. Contentedness (post)	0.077	0.306	0.475 *	0.743 **	0.865 **	0.739 **	0.241	1	0.023	0.182	0.182	0.318	0.019
9. Mu (μ)	0.399	0.388	0.242	0.291	0.302	−0.038	0.155	0.182	1	0.691 **	−0.004	0.181	−0.026
10. Sigma (σ)	0.491 *	0.459 *	0.388	0.433	0.371	−0.134	0.155	0.182	0.691 **	1	0.108	0.258	−0.032
11. Tau (τ)	0.118	0.096	0.104	0.290	0.188	−0.117	−0.149	0.182	−0.004	0.108	1	0.531 *	−0.247
12. Hits	0.001	0.345	−0.091	0.323	0.388	0.195	−0.221	0.318	0.181	0.258	0.531 *	1	−0.120
13. P3a ERP	−0.227	−0.310	−0.251	0.138	−0.095	0.015	0.275	0.019	−0.026	−0.032	−0.247	−0.120	1

Note: * *p* < *0*.05, ** *p* < *0*.01.

## Data Availability

The data supporting the findings of this study are available via the Northumbria Figshare repository.
